# Myotendinous junction tear of the anterior bundle of the supraspinatus muscle—a rare pattern of injury involving rotator cuff muscles

**DOI:** 10.1259/bjrcr.20200004

**Published:** 2020-07-15

**Authors:** Ana C. Vieira, Esther Montez Pérez, Moises F. Hernando, Faustino Abascal, Luis Cerezal

**Affiliations:** 1 Hospital do Espirito Santo, Évora, Portugal; 2 Diagnostico Medico Cantabria, Santander, Spain

## Abstract

Myotendinous junction injuries are rare and often present with distinctive imaging findings that should be differentiated from purely tendinous degenerative ruptures.

Myotendinous junction tears are common in the lower limb but rarely involve rotator cuff muscles. Considering rotator cuff muscles, the infraspinatus and supraspinatus muscles are the most frequently implicated.

The intrinsic anatomy of the supraspinatus muscle gives it a greater contractile force and consequently a propensity for rupture. It is composed of two bundles: anterior and posterior (with the latest further divided in a deep anterior, a medial and a superficial posterior portion). These two components have distinctive anatomy with the anterior bundle having a long intramuscular tendon and bipennate configuration and the posterior bundle having a smaller intramuscular tendon and parallel muscle fibres. This distinctive anatomy grants a greater contractile force to the anterior bundle of the supraspinatus muscle and for this reason it is more prone to myotendinous rupture.

This type of injury has been associated with a rapid progression to severe fatty infiltration and should be differentiated from purely tendinous tears that are more frequent and associated with degenerative changes. Myotendinous tears occur centrally located in the muscle belly and are not associated with full thickness tears of the distal tendon attachment.

## Clinical presentation

57-year-old male felt shoulder pain and an audible pop on his left shoulder while performing an abrupt abduction and external rotation movement. He was seen by an orthopaedic surgeon who requested a magnetic resonance (MR) of the left shoulder.

## Differential diagnosis

1. partial thickness bursal-sided supraspinatus tendon tear
2. subacromial impingement syndrome
3. full-thickness supraspinatus tear
4. subacromial/subdeltoid bursitis

## Imaging findings

**Figure 1.**
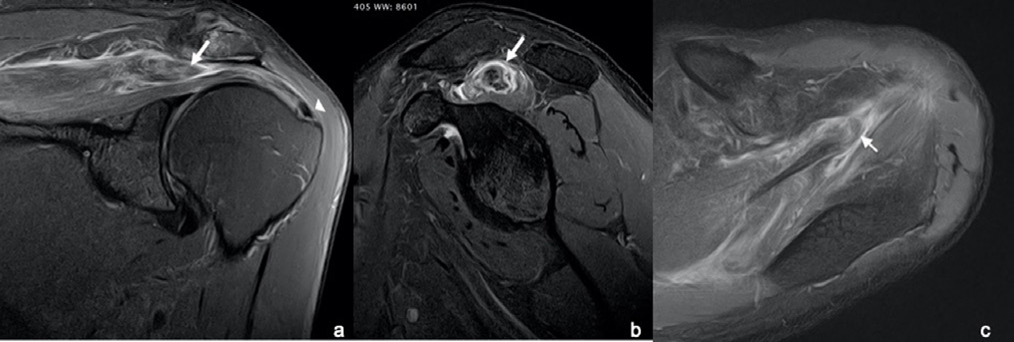
T2 fat sat-weighted images of the shoulder: a) coronal, (b) sagittal and c) axial planes of the shoulder demonstrating a full-thickness, selective (arrows), myotendinous junction complete rupture of the anterior supraspinatus bundle. The deep anterior, medial and superficial posterior portions of the posterior bundle of the supraspinatus muscle are preserved as well as the tendinous attachment of the anterior bundle at the humeral head (arrow head).

**Figure 2.**
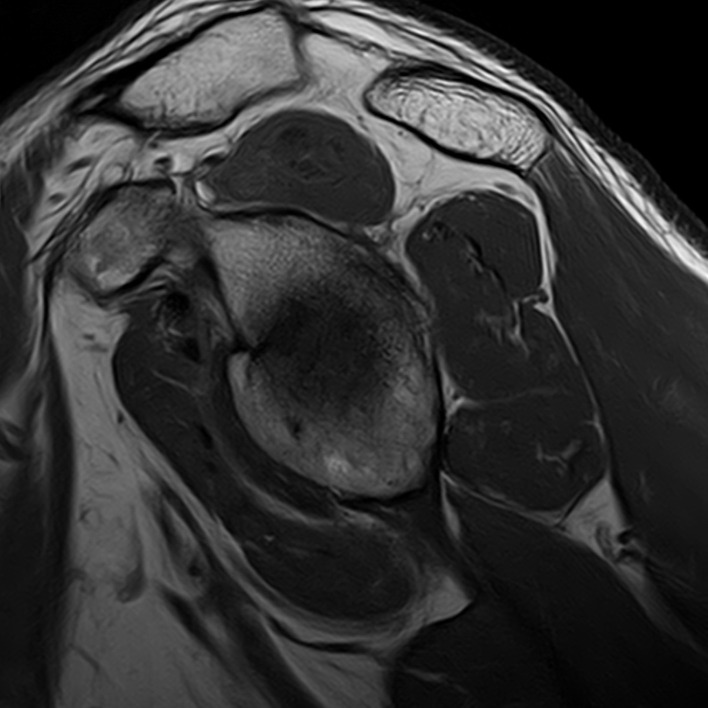
T1W image in sagittal plane demonstrating the trophicity of the rotator cuff muscles—a preserved muscle trophicity was found, supporting an acute onset.

**Figure 3.**
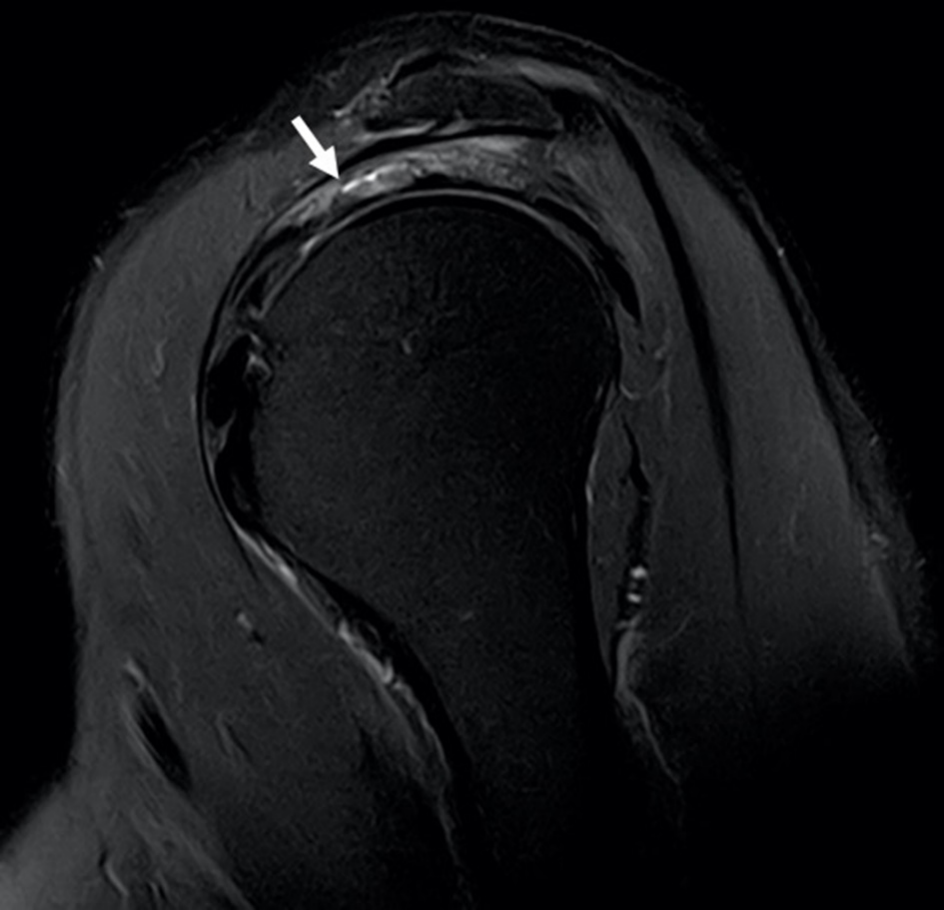
T2 fat sat-weighted image of the shoulder in sagittal plane. Distortion and hyperintense signal of intratendinous tendon of anterior supraspinatus muscle with surrounding oedema (arrow) consistent with myotendinous injury. No degenerative tendinosis of the posterior bundle of the supraspinatus tendon was observed nor concurrent tears/degenerative changes of the other rotator cuff tendons was noted.

**Figure 4.**
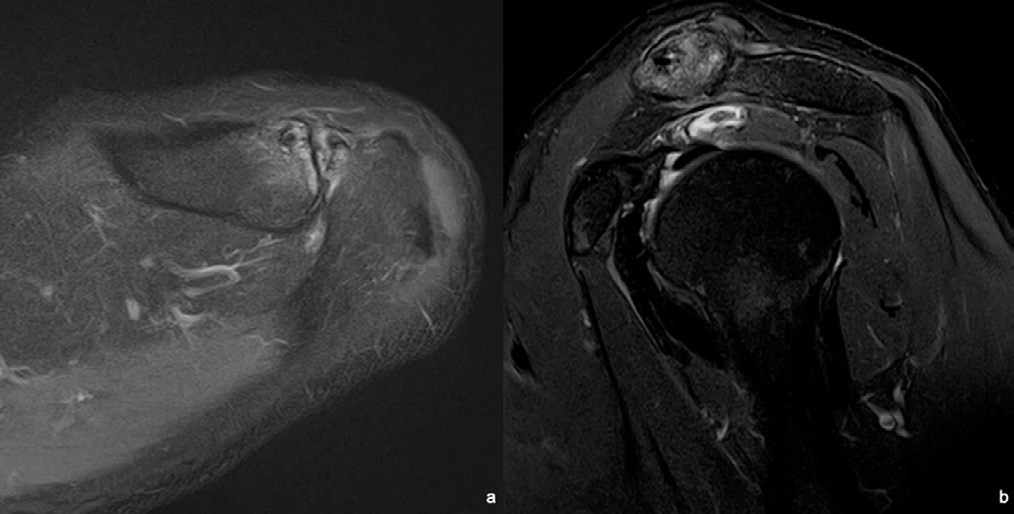
T2W fat sat images of the acromio-clavicular joint: a) axial and b) sagittal views of the acromio-clavicular joint showing moderate degenerative arthropathy without significant indentation of the medial portion of the subacromial space.

A MR study was performed in a 3T machine using a dedicated shoulder coil. The study revealed a full-thickness rupture of the anterior bundle of the supraspinatus muscle at the level of the myotendinous junction with associated oedema and muscle retraction to the level of the acromio-clavicular joint (Figure 1a, b and c). It is also appreciated intramuscular oedema and reactive bursitis of the subacromial/subdeltoid bursa (Figure 1a). Muscle trophicity was preserved, supporting an acute onset (Figure 2). No concomitant degenerative tendinosis of the posterior bundle of the supraspinatus tendon was seen (Figure 3). Moderate degenerative arthropathy of the acromio-clavicular joint was seen but without significant indentation of the supraspinatus space (Figure 4a and b). There were no other concomitant rotator cuff tears and no humeral head subluxation was present.

## Treatment

No consensus was found on how to treat this type of rotator cuff ruptures (conservative *vs* surgical) as they both usually progress to severe fatty infiltration.

## Outcome, follow-up

Using MR, we can grade myotendinous injuries in terms of severity which correlates with patient outcome. Grade I injuries correspond to muscle stretch which usually heal without sequelae, grade II injuries are partial ruptures in which there is no tendon retraction and grade III injuries are complete ruptures of the musculotendinous junction as illustrated in the case presented and usually associated with a poor outcome if treated conservatively.^1^ Consequently, an accurate diagnosis is needed specially because, they may be overlooked arthroscopically as they are extraarticular in nature.^2^ Furthermore, this particular type of tear is associated with a rapid progression to severe fatty infiltration.^1,3^

## Discussion

Rotator cuff tears are one of the most common pathologies of the shoulder and one of the commonest causes of shoulder pain.^4^ They can be associated with different mechanisms of injury as: trauma, subacromial impingement or tendon degeneration.^5^

Most commonly seen rotator cuff tears are present at the level of the tendinous insertion to the bone in its footprint in the extra muscular part of the tendon.^3,6^ They can be divided into partial thickness (bursal, articular sided or interstitial tears) or complete/full-thickness tears. The latest group can be associated with different degrees of tendinous retraction.^7,8^

Patte classification is now widely used for this purpose with the degree of retraction being divided in three grades:

1. Grade 1—proximal stump near bony insertion,
2. Grade 2—proximal stump at the level of the humeral head,
3. Grade 3—proximal stump at the level of glenoid or proximal to it.

Rotator cuff myotendinous junction injuries are distinct from the commonly seen rotator cuff degenerative tendon tears regarding location, epidemiology, radiological characteristics and outcome.^2^

In fact, the myotendinous junction is the frailest location of the myotendinous unit as it is less capable to resist to force absorbing energy.^3^

Myotendinous junction ruptures occur when a contracted muscle is subjected to a motion of forceful uncontrolled elongation during a phase of eccentric muscle contraction. This type of lesions are commonly seen and well recognized in lower limbs being rare in superior limbs (average of 0.26–0.71 cases per year) and often forgotten.^2^

Some intrinsic characteristics are proven to favour ruptures at the level of the myotendinous junction such as pennate muscle architecture, large tendon that crosses two joints, intramuscular tendon component and the capability to produce great contractile forces.

The supraspinatus muscle share some of these characteristics. It is composed of two bundles: an anterior and a posterior bundle that can be further divided into a deep anterior, a medial and a superficial posterior portion. These two components have distinctive anatomy with the anterior bundle with a long intramuscular tendon and bipennate configuration and the posterior bundle with a smaller intramuscular tendon and parallel muscle fibres. This distinct anatomy confers a greater contractile force in the anterior bundle of the supraspinatus muscle and for this reason it is more prone to myotendinous rupture.^2,9,10^

On MR, it is possible to accurately identify the myotendinous junction and its injury as the signal intensity abnormality and fibres discontinuity will be centred around the myotendinous junction, rather than at the tendon attachment (as in the most commonly seen tendon tears).^3,11^

In conclusion, radiologists must be aware of the particular anatomy of the rotator cuff muscles and its implications being able to identify this rare type of superior limb injury.

## Learning points

1. Myotendinous rupture injuries of the rotator cuff muscles are rare.
2. The anatomic characteristics of the supraspinatus muscle make it one of the most prone to this type of injury in the upper limb, in particular its anterior bundle.
3. On MR, it is possible to accurately identify a myotendinous junction and its injury as the signal intensity abnormality on fluid sensitive sequences and fibre discontinuity will be centred around the myotendinous junction.
4. This particular type of tear is associated with a rapid progression to severe fatty infiltration.
5. Therapeutic approach (surgical *vs* conservative) remains controversial.
